# Lichen Planus Following COVID‐19 Infection and Vaccination. Matched Case–Control Study

**DOI:** 10.1111/ajd.14522

**Published:** 2025-05-05

**Authors:** Paolo Giacomo Arduino, Alexey Kubanov, Anna Vlasova, Andrey Martynov, Stefano Petti

**Affiliations:** ^1^ Department of Surgical Science University of Turin Turin Italy; ^2^ State Research Center of Dermato‐Venereology and Cosmetology Moscow Russian Federation; ^3^ Sechenov First Moscow State Medical University Moscow Russian Federation; ^4^ Department of Public Health and Infectious Diseases Sapienza University Rome Italy

**Keywords:** COVID‐19, infections, lichen planus, SARS‐CoV‐2, vaccination

## Abstract

**Background:**

New‐onset lichen planus (LP) development following COVID‐19 infection/vaccination is reported. Since case series cannot be used to study exposure–outcome associations, we designed this matched case–control study to investigate whether COVID‐19‐related events and de novo LP are associated.

**Methods:**

Patients with histologically confirmed LP, newly diagnosed at the National Research Center of Dermato‐Venereology of Moscow (September 2020–December 2022), were considered. Sex/age/ethnicity‐matched controls attending the same Center in the same period for consultations on conditions unrelated to LP were selected. Cases/controls with known LP trigger factors were excluded. COVID‐19‐related events were: symptomatic COVID‐19 (PCR‐confirmed), and COVID‐19 vaccination (viral vector vaccine) occurred ≤ 1 month before the visit at the Center. The association between COVID‐19‐related events and LP was assessed with conditional (Mantel–Haenszel method) and unconditional (logistic regression analysis adjusted for sex, age, and smoking) analyses. Subgroup analysis, with COVID‐19 infection and vaccination treated separately, and sensitivity analysis on another group of patients with suspected LP, not confirmed histologically, were also made.

**Results:**

Fifty‐five case–control pairs were considered. Mean age (51 years), sex (56.4% females) and ethnicity (100% whites) distributions were the same in both groups. Conditional and unconditional odds ratios resulted 7.50 (95% Confidence Interval –95CI, 1.72–32.80), 4.45 (95CI, 1.63–12.15), respectively (*p* < 0.05). Subgroup analysis confirmed the association between symptomatic infection and LP, while sensitivity analysis corroborated the results of the primary analysis.

**Conclusions:**

This observational study, reporting a strong significant association between COVID‐19 infection/vaccination and de‐novo LP, suggests that COVID‐19‐related events, especially infection, could act as LP trigger factors.

## 1 Introduction

Lichen planus (LP) is a T cell‐mediated autoimmune disease with an estimated prevalence of 0.4% in adults [1]. Although its aetiology is unknown, LP development may be triggered by viral infections, such as Hepatitis C virus, Human Papillomavirus, Herpesviruses, and medications, including vaccines against Hepatitis C virus, Human Papillomavirus, and Influenza virus [2, 3, 4, 5, 6]. During the recent COVID‐19 pandemic, several case reports and series showed the development of LP between one and sixty days after COVID‐19 infection and vaccination [7, 8, 9, 10]. These studies suggested the hypothesis that COVID‐19 and LP could be associated [11].

This hypothesis is not confirmable with case reports and series, due to the lack of comparison groups who developed LP and did not experience COVID‐19 infection/vaccination [12]. In addition, the golden standard of epidemiologic studies, that is, clinical trials and, secondarily, cohort studies, is unfeasible. Indeed, with an annual incidence rate of 20‐30 × 100,000 adults [13, 14], these studies require sample sizes as large as one million individuals to follow for more than 1 year. The matched case–control study is, therefore, the most appropriate study type to investigate LP [15], as it does not require a large sample and can control for unquantifiable and unknown confounding factors [16].

For this reason, this matched case–control study was designed to investigate the association between COVID‐19 infection/vaccination and de novo LP development.

## 2 Methods

### 2.1 Study Design

Cases were patients referred to the National Research Center of Dermato‐Venereology and Cosmetology of Moscow (Russia), who received a new diagnosis of LP; controls were patients referred to the same Center for diseases that did not share trigger factors, signs, and symptoms with LP. Controls were matched with cases by age (±5 years), sex, and ethnicity. Case‐to‐control ratio was 1:1. The exposure variable included symptomatic COVID‐19 and COVID‐19 vaccination occurring up to 1 month before the first visit at the Center. LP exacerbations and recurrences were not considered, because their assessment could be influenced by personal opinions and psychological traits of patients and clinicians, and could be subjected to diagnostic bias, while the diagnosis of de novo LP, requiring clinical and histopathological evaluations, was considered more dependable [15].

### 2.2 Selection of Cases and Controls

New LP cases (ICD‐10 codes, L43.0, L10.1, L43.3, L43.8, L43.9), referred to the Center (September 2020–December 2022) were eligible. Patients with an LP diagnosis made in other healthcare settings were not considered. LP diagnosis was made by trained and expert dermatologists (A.K., and A.M.) and was based on clinical cutaneous and mucosal manifestations and skin biopsy for histopathologic confirmation. Patients with lichenoid drug reaction (L43.2) and other papulosquamous disorders (L44) were excluded [17].

Controls were recruited among patients attending the same Center for consultations on conditions such as nevus, papilloma, and lipoma. Each matched control was selected among patients admitted to the Center in the same period (±3 months) as the corresponding case. In order to exclude known LP trigger factors, case and control patients were not under routine pharmacologic therapies during the month preceding the first visit at the Center, did not receive a diagnosis of HIV, serious herpesvirus infections, cancer, and haematological or autoimmune diseases, and had not laboratory confirmed HCV infection.

### 2.3 Exposure

Several studies found that several cutaneous manifestations following SARS‐CoV‐2 infection and various types of COVID‐19 vaccination were clinically similar [18, 19], suggesting molecular mimicry of COVID‐19 vaccines with the viral epitope present on the Spike (S) protein of SARS‐CoV‐2, and/or delayed T‐cell mediated hypersensitivity response to vaccination, that would be similar to the response to infection [18, 20]. For this reason, symptomatic COVID‐19 infection and COVID‐19 vaccination with the viral vector vaccine used in Russia, that contained the gene encoding the full‐length S protein [21], were considered as a single type of exposure variable, called “COVID‐19 related event”.

Data on symptomatic COVID‐19 and vaccination administration during the month preceding the first visit to the Center were gathered and were confirmed by the Federal Service for the Oversight of Consumer Protection and Welfare, which was the COVID‐19 surveillance body during the pandemic. Symptomatic COVID‐19 was confirmed by PCR positive test [22]. The time elapsed between infection confirmation/last vaccination and first presentation to the Center was assessed.

### 2.4 Ethics Approval and Informed Consent

The study protocol was approved by the Ethical Committee of the National Scientific Center for Dermato‐Venereology and Cosmetology of the Russian Ministry of Health (approval #298, 29 February 2024). This study was conducted in accordance with the Helsinki declaration.

The patients in this manuscript have given their written informed consent to participate in the study. Complete anonymity was guaranteed by using data cumulatively without details of individual cases.

### 2.5 Statistical Analysis

The McNemar's test was used to assess the sample size. Without similar studies ever published, the proportion of discordant pairs (i.e., the proportion of case/matched‐control couples with different exposure status) was set at 30%, and the odds ratio conditional on the discordance (i.e., the ratio between discordant pairs) at 5.0. Alpha and beta errors were set at 0.05 and 0.20, respectively. The minimum required number of case–control pairs was 52.

The differences between case and control groups in mean age and in frequency distributions according to sex, ethnicity, smoking status, number of individuals who had any COVID‐19 related events, infection, and vaccination were initially tested with the Student's t (differences between means) and the standard *χ* ^2^ test (differences between proportions). Then, the differences in the proportion of the exposed in the two groups was assessed with the McNemar's test. The association between exposure to COVID‐19 and de novo LP development was investigated with conditional and unconditional analyses. The Mantel–Haenszel method unadjusted for confounders was used for the conditional analysis, and the pair‐matched odds ratio (OR) with 95% confidence interval (95CI) was assessed. Unconditional logistic regression analysis adjusted for age, sex, ethnicity, and smoking status was performed [16]. Other confounding factors, such as the presence of autoimmune diseases, cancer, pharmacological therapies, and HCV infection, that could act as LP trigger factors were controlled, as eligible cases and controls with these characteristics were excluded during the sample enrolment stage. Collinearity was investigated with Pearson's r correlation matrix that included all the explanatory variables (the variable ‘age’ was dichotomized into lower/higher than the average age of the sample). If highly correlated variables were detected (*r* ≥ 0.5), their interaction term was included in the multiple regression model.

Subgroup analysis was made, splitting the COVID‐19 related events into two different variables, namely, symptomatic COVID‐19 and COVID‐19 vaccination. Multiple logistic regression analysis was repeated, including these two exposure variables.

Sensitivity analysis was made. Presumptive LP cases, that is, subjects who had only a clinical LP profile without histopathologic confirmation, were considered and were age (±5 years), sex, and ethnicity matched with controls, and the analyses were repeated.

This study was limited to the period 2020–2022, because the COVID‐19 surveillance was eased following the declaration of the end of the pandemic in 2023 [23]. Thus, starting from 2023, the probability of information bias [16], due to subjects who actually had COVID‐19 and did not undergo the SARS‐CoV‐2 testing, or subjects who could not recall whether and when they were vaccinated, was high.

The statistical analyses were made using MedCalc (MedCalc Software, version 20_1), StatView (SAS Institute, version 5.0.1), OpenEpi version 3.01.

## 3 Results

Fifty‐nine patients were diagnosed with de novo LP during the study period; four were excluded, three with cancer, and one who was HIV positive, thus leaving 55 pairs for the analysis. LP frequency distribution according to ICD‐10 is in Table 1; nine patients had cutaneous and oral LP. Mean age of cases and controls was 51 years (Table 2); females were prevalent, and all patients were whites. Cases reported significantly more COVID‐19 related events in the month preceding the examination than controls (38.2% vs. 14.5%). Infection (23.6% vs. 5.4%) and vaccination (18.2% vs. 9.1%) were more frequent among cases, but the χ^2^ test was statistically non‐significant for vaccination.

**TABLE 1:** Frequency distribution of lichen planus cases according to ICD‐10 classification.

ICD‐10 code	ICD‐10 definition	% (number)	Oral lichen planus
L43.0	Hypertrophic LP	1.8% (1)	11.1% (1)
L43.1	Bullous LP	3.6% (2)	0
L43.3	Subacute (active) LP—LP tropicus	5.5% (3)	11.1% (1)
L43.8	Other LP	50.9% (28)	33.3% (3)
L43.9	LP, unspecified	38.2% (21)	44.4% (4)

Abbreviation: LP, lichen planus.

**TABLE 2:** General characteristics of cases (patients with de‐novo LP) and matched controls. Statistical analysis of differences between cases and controls (standard *χ* ^2^ test –corrected for continuity in 2 × 2 tables, differences between proportions; Student's *t*‐test–paired samples for age, differences between means).

	Cases (*N* = 55)	Controls (*N* = 55)	Statistical analysis
Mean age	51.2 ± 15.4	50.7 ± 15.4	*t* = 1.41; *p* = 0.16
Age range	28–89	24–86	
Sex			*χ* ^2^ = 0.00; *p* > 0.99
Females	56.4% (*N* = 31)	56.4% (*N* = 31)	
Males	43.6% (*N* = 24)	43.6% (*N* = 24)	
Ethnicity			
Whites	100.0% (*N* = 55)	100.0% (*N* = 55)	*χ* ^2^ = 0.00; *p* > 0.99
Current smoker	27.3% (*N* = 15)	32.7% (*N* = 18)	*χ* ^2^ = 0.17; *p* = 0.67
COVID‐19 related event^a^	38.2% (*N* = 21)	14.5% (*N* = 8)	*χ* ^2^ = 6.74; *p* = 0.009
Symptomatic COVID‐19^b^	23.6% (*N* = 13)	5.5% (*N* = 3)	*χ* ^2^ = 5.92; *p* = 0.01
COVID‐19 vaccination^b^	18.2% (*N* = 10)	9.1% (*N* = 5)	*χ* ^2^ = 1.23; *p* = 0.26
COVID‐19 vaccine dose			*χ* ^2^ = 0.37; *p* = 0.82
First	30.0% (*N* = 3)	20.0% (*N* = 1)	
Second	60.0% (*N* = 6)	60.0% (*N* = 3)	
Third	10.0% (*N* = 1)	20.0% (*N* = 1)	
Interval between COVID‐19 related event and first visit	16.3 ± 7.4	14.8 ± 6.9	*t* = 0.52; *p* = 0.60

^a^ COVID‐19 infection or vaccination occurred/administered ≤ 1 month before the first visit at the Center.

^b^ Occurred/administered ≤ 1 month before the visit at the Center.

The conditional OR for LP development was 7.50 (95CI, 1.72–32.80; *p*=0.003), while the adjusted OR was 4.45 (95CI, 1.63–12.15; *p*=0.003). These results were corroborated by the McNemar's test (Table 3). No relevant collinearity was found among the explanatory variables, but there was a statistically significant correlation between COVID‐19 related events and age (*r* =−0.319; *p*=0.0006) (Table S1). The inclusion of this interaction term in the logistic regression model slightly changed the odds ratio estimate (OR 4.10; 95CI, 1.33–12.65; *p*=0.01; data not in Table).

**TABLE 3:** Association between COVID‐19 related events (symptomatic infection and vaccination) occurred less than 1 month before the first visit at the Center and de‐novo LP development.

	odds ratio	95% confidence interval	*p*
Conditional, unadjusted	7.50	1.72–32.80	0.003
Unconditional, adjusted^a^	4.45	1.63–12.15	0.003

*Note:* McNemar's test corrected for continuity: 8.47, *p* = 0.003 (COVID‐19 related events); 5.78, *p* = 0.01 (symptomatic COVID‐19); 1.45, *p* = 0.22 (COVID‐19 vaccination).

^a^ Logistic regression analysis adjusted for age, sex, smoking status.

The sample size was not large enough for a reliable subgroup analysis; thus, non‐significant associations were expected. Nevertheless, such an association resulted strong for COVID‐19 infection, with OR=6.84 (95CI, 1.70–27.54; *p*=0.006), and marginally non‐significant for vaccination (OR=2.85; 95CI, 0.82–9.83; *p*=0.09) (Table 4).

**TABLE 4:** Subgroup analysis. Association between symptomatic COVID‐19 and COVID‐19 vaccination occurred less than 1 month before the first visit at the Center and de novo LP development.

	odds ratio	95% confidence interval	*p*
Symptomatic COVID‐19	6.84	1.70–27.54	0.006
COVID‐19 vaccination	2.85	0.82–9.83	0.09

*Note:* Logistic regression analysis adjusted for age, sex, smoking status.

The sensitivity analysis included 98 patients with histologically unconfirmed LP diagnosis and their matched controls; their mean age was 50 years. Once again, COVID‐19 related events, particularly infection, were significantly more frequent in presumptive cases than in controls (Table S2). The conditional and unconditional ORs for de novo LP development were not significantly different from the ORs resulting from the primary analysis (Table S3).

## 4 Discussion

This study found a strong association between COVID‐19 related events and de novo LP development. Many cutaneous conditions following COVID‐19 are reported, such as alopecia areata [24], pemphigus foliaceus and vulgaris [12], erythema multiforme [25, 26], Herpes Zoster [27], and LP [8], some of which are thought to be causally associated with COVID‐19 related events [11, 28]. However, the development of these conditions after COVID‐19 related events does not automatically mean that COVID‐19 infected and/or vaccinated individuals are at higher risk of these manifestations [29, 30]. Indeed, case series and reports describe the temporal sequence of two events, but do not have the potential to investigate whether these events are associated.

The present results are corroborated by two studies that used large population databases. Namely, the ORs for LP in patients registered at the University of Florida Healthcare Centers were 1.1 (*p* = 0.52) and 1.6 (*p* < 0.001) after COVID‐19 infection and vaccination, respectively [31], while the OR for oral lichenoid lesions/oral LP after COVID‐19 vaccination in patients registered at the TriNetX Global Health Research Network (Cambridge, Massachusetts) was 2.5 (*p* < 0.0001) [32]. These studies, however, could be biased, due to the so called ‘healthy user effect’ [33], as individuals who are vaccinated or tested for SARS‐CoV‐2 –a precondition for COVID‐19 diagnosis, differ in the general health status and in other characteristics than those who are not. Therefore, the exposed and unexposed individuals are not comparable. In the reported studies, for example, vaccinated individuals could be more aware of cutaneous reactions and seek for medical advice more often than the unvaccinated. Therefore, the higher number of LP cases diagnosed in the vaccinated patients would be apparent and explained by their proneness to seek for medical advice.

The present study has limits. First, cases and controls were not extracted from the general population, but from patients referred to the Dermato‐Venereology and Cosmetology Center. Therefore, it is not possible to conclude that the same strong association between COVID‐19 and LP, found in the present sample of patients, also was true among subjects who developed LP during the study period, but did not seek for healthcare advice, a problem known as selection bias. Second, information bias could be due to unapparent exposure, as cases and controls may have developed asymptomatic SARS‐CoV‐2 infection that could be undetected, and if the proportion of subjects with undetected infection was higher in cases than in controls, then the reported infection/LP association would be weaker or nullified. However, although this bias could be possible, its impact on the results was limited, as access to the Center for routine visits was allowed only to patients providing recent negative PCR tests, and this situation applied to both cases and controls. Third, patients with new onset LP seeking healthcare at the Center could be more keen to recall what may have caused their illness and be better motivated to remember details of COVID‐19‐related events than the controls. This form of information bias did not affect the results of this study, as data on vaccination and symptomatic infection were confirmed by the Federal Service for the Oversight of Consumer Protection and Welfare, and the study was interrupted when COVID‐19 surveillance was eased.

There could be differences in the access to healthcare between patients eligible as cases and those eligible as controls. In the early pandemic stages, the Russian healthcare system was reorganised to provide care to COVID‐19 patients, maintaining essential services and reducing planned medical care, while starting from June 2020, the healthcare services gradually returned to normal activities, and in September 2020, the demand for medical services counterbalanced the reduced activity of previous months [34, 35]. Although several Dermatology clinics were temporarily closed, the National Research Center of Dermato‐Venereology and Cosmetology remained available for all outpatient services, excluding aesthetic surgery. For this reason, the number of outpatient visits to the Center in 2020 almost doubled compared to 2019 (23,031 vs. 12,758). This suggests that both patients eligible as cases and those eligible as controls had similar opportunities to attend the Center, and the potential impact of an ‘admission‐to‐healthcare’ bias was probably minimal.

There could be differences between individuals eligible as cases and those eligible as controls in the demand for healthcare following COVID‐19 infection and vaccination, as LP patients often show peculiar psychological traits, such as anxiety, negative well‐being, depressed mood, and low self‐control [36]. Therefore, individuals with these traits who had COVID‐19 or were vaccinated against SARS‐CoV‐2, and noticed the development of lesions suggestive of LP, could be more likely to seek dermatological advice than individuals eligible as controls, because they could be afraid of COVID‐19 complications and vaccine adverse events. In order to limit the impact of this potential bias, only patients with de novo LP were considered in the present study, assuming that the appearance of flares and disease exacerbations after infection or vaccination in anxious patients with previous LP diagnosis could induce them to seek for dermatological advice. The impact of this bias in patients with de novo LP is likely minimal, as confirmed by a study from a Chinese Dermatology outpatient department, with similar admission policies as in the National Research Center of Dermato‐Venereology, that did not report an increase in new LP diagnoses in 2021 compared to 2019 [37].

Causal inference on the potential association between COVID‐19 and de novo LP development must be careful, due to the limits of studies that are not clinical trials [38, 39, 40]. The reported strong statistical association between these events, corroborated by case report/series [7, 8, 9, 10], and large population database studies [31, 32], along with a sort of presumptive biological plausibility, implying molecular mimicry and/or delayed T‐cell mediated hypersensitivity reaction of vaccines [11, 18, 19, 20], suggest that this association could be causal. However, for LP and other autoimmune diseases, the concept of causation must be interpreted cautiously. Namely, although de novo LP could be diagnosed following events such as microbial infections and vaccinations, these events are not the real cause of LP [41, 42, 43], they are trigger factors of this disease. The concept of trigger factor cannot be included in any of the various categories of cause [40], however, LP and other autoimmune diseases cannot be diagnosed without the intervention of a trigger factor, and it is imaginable that if all the LP trigger factors were known and removed, this disease would no longer be diagnosed in predisposed individuals. But trigger factors are innumerable and it is impossible to remove them all. Thus, although predisposed individuals have developed LP following COVID‐19 infection/vaccination, they would likely develop it in their lifetime even without COVID‐19 related events, due to the intervention of other trigger factors.

In conclusion, in the present sample of adult patients seeking healthcare in a large specialist dermatology clinic, COVID‐19 related events were significantly associated with de novo LP development, thus tentatively suggesting the hypothesis that these events could act as LP trigger factors.

## Ethics Statement

Ethics approval was granted on 29 February 2024 by the Ethical Committee of the National Scientific Center for Dermato‐Venereology and Cosmetology of the Russian Ministry of Health (available in Russian upon request).

## Consent

The patients in this study have given their written informed consent to participation. Complete anonymity was guaranteed by using data cumulatively without details of individual cases.

## Conflicts of Interest

The authors declare no conflicts of interest.

## Supporting information


Data S1.

## Data Availability

The data that support the findings of this study are available on request from the corresponding author. The data are not publicly available due to privacy or ethical restrictions.
