# Supplementary material for: Lichen Planus Following COVID‐19 Infection and Vaccination. Matched Case–Control Study
Source: Australas J Dermatol. 2025 May 5;66(5):289–95. doi: 10.1111/ajd.14522 (PMC12334811; doi:10.1111/ajd.14522)
Supplement: Supplementary file 1 — Data S1. [file AJD-66-289-s001.docx]

**Supplementary TABLE 1**

Collinearity (Pearson’s correlation coefficient, r) between the explanatory variables used for the logistic regression analysis displayed in Table 3. Namely, “any COVID-19 related event” (no 0, yes 1), “age” dichotomized into lower than/equal to or higher than the average age of 51 years (<51 years 0, ≥51 years 1), “sex” (males 0, females 1), “smoking status” (past/no smoker 0, current smoker 1). Pearson’s correlation coefficients (r). Collinearity was considered relevant for coefficients higher than 0.5

| variable | | r (p value) |
| --- | --- | --- |
|  |  |  |
| any COVID-19 related event | age | -0.319 (0.0006) |
| any COVID-19 related event | sex | -0.139 (0.14) |
| any COVID-19 related event | smoking status | -0.032 (0.74) |
| age | sex | 0.393 (<0.0001) |
| age | smoking status | -0.103 (0.28) |
| sex | smoking status | -0.224 (0.01) |

**Supplementary TABLE 2**

General characteristics of patients with clinical diagnosis of de-novo LP without histologic confirmation and their matched controls. Statistical analysis of differences between cases and controls (standard χ^2^ test –corrected for continuity in 2x2 tables, differences between proportions; Student’s t-test –paired samples for age, differences between means)

|  | cases (N=98) | controls (N=98) | statistical analysis |
| --- | --- | --- | --- |
|  |  |  |  |
| Mean age | 50.0±15.8 | 50.3±15.1 | t=1.02; p=0.30 |
| Age range | 28-89 | 24-86 |  |
| Sex |  |  | χ^2^=0.00; p>0.99 |
| Females | 61.2% (N=60) | 61.2% (N=60) |  |
| Males | 38.8% (N=38) | 38.8% (N=38) |  |
| Ethnicity |  |  |  |
| Whites | 100.0% (N=98) | 100.0% (N=98) | χ^2^=0.00; p>0.99 |
| Current smoker | 29.6% (N=29) | 25.5% (N=25) | χ^2^=0.23; p=0.63 |
| COVID-19 related event † | 36.7% (N=36) | 17.3% (N=17) | χ^2^=8.37; p=0.003 |
| Symptomatic COVID-19 ‡ | 23.5% (N=23) | 10.2% (N=10) | χ^2^=5.24; p=0.02 |
| COVID-19 vaccination ‡ | 13.3% (N=13) | 8.2% (N=8) | χ^2^=0.85; p=0.35 |
| COVID-19 vaccine dose ‡ |  |  | χ^2^=1.21; p=0.54 |
| First | 7.7% (N=1) | 25.0% (N=2) |  |
| Second | 61.5% (N=8) | 50.0% (N=4) |  |
| Third | 30.8% (N=4) | 25.0% (N=2) |  |
| Interval between COVID-19 related event and first visit | 16.4±6.5 | 17.6±6.1 | t=0.62; p=0.53 |

†COVID-19 infection or vaccination occurred/administered less than one month before the first visit at the dermatovenereology center

‡occurred/administered ≤1 month before the visit at the dermatovenereology center

**Supplementary TABLE 3**

Sensitivity analysis. Association between COVID-19 related events occurred less than one month before the first visit at the Center and de-novo LP development in patients with clinical LP diagnosis without histologic confirmation and their matched controls (98 pairs)

|  | odds ratio | 95% confidence interval | p-value |
| --- | --- | --- | --- |
|  |  |  |  |
| Conditional, unadjusted | 3.11 | 1.47-6.59 | 0.003 |
| Unconditional, adjusted † | 2.94 | 1.48-5.86 | 0.002 |

McNemar’s test corrected for continuity: 8.75, p=0.003 (COVID-19 related events); 4.64, p=0.03 (symptomatic COVID-19); 0.94, p=0.33 (COVID-19 vaccination)

†logistic regression analysis adjusted for age, sex, smoking status.
